# Supplementary material for: Physical modeling of ribosomes along messenger RNA: Estimating kinetic parameters from ribosome profiling experiments using a ballistic model
Source: PLoS Comput Biol. 2023 Oct 20;19(10):e1011522. doi: 10.1371/journal.pcbi.1011522 (PMC10659217; doi:10.1371/journal.pcbi.1011522)
Supplement: S1 Fig — (PDF) [file pcbi.1011522.s001.pdf]

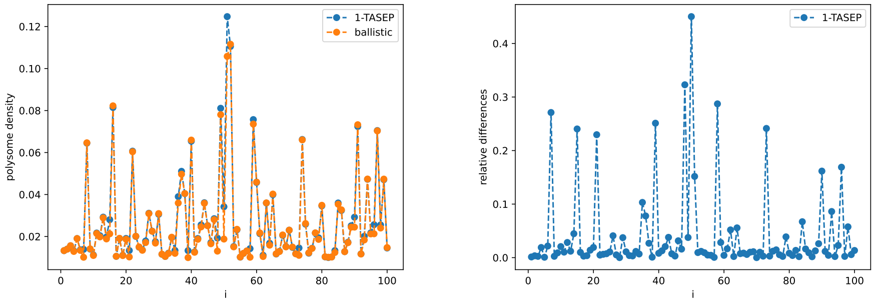

Figure 1: **Quantifying the effect of excluded volume interaction in the ballistic model.**(left) We plot the polysome profile as a function of the genomic coordinate expressed in codon unit i ($L = 100$). We use typical biological parameters $\alpha = 0.06s^{-1}$ and $\theta = 1/\omega = 60$min. Elongation rates are obtained from a uniform distribution in $[0.5s^{-1}; 6s^{-1}[$. The ballistic model (orange) is compared to Monte Carlo simulations of the TASEP model (blue). The simulations are performed using the Gillespie algorithm and the number of independent samples is 2.7×10^6. As shown in the main text, these parameters tune the system in the intermediate degradation regime for the k-some profiles, in which no effect are visible on polysomes. We notice that the two curves are in good agreement except in some specific genomic coordinates. (right) The absolute relative difference between the two densities on the left panel is plotted versus the codon. This error is defined as $\Delta = \frac{|\rho_{TASEP}(i) - \rho_{ballistic}(i)|}{\rho_{TASEP}(i)}$. The relative error Δ between the two models is never larger than 5% percent of the signal, except in specific combination of sites (a fast codon followed by a slow codon) where correlation become stronger n a fast codon (i.e. low density) will be followed by a slow codon (i.e. large density). The resulting bottleneck results in a traffic jam of ribosome in the TASEP model, which cannot be accounted for in the ballistic model. As a result, the density of the TASEP will be higher than the ballistic model before the slow codon and will be lower at the slow codon. Therefore the ballistic model is a very good approximation of the TASEP model within *homogeneous* stretches of hopping rates.
