# Supplementary material for: Physical modeling of ribosomes along messenger RNA: Estimating kinetic parameters from ribosome profiling experiments using a ballistic model
Source: PLoS Comput Biol. 2023 Oct 20;19(10):e1011522. doi: 10.1371/journal.pcbi.1011522 (PMC10659217; doi:10.1371/journal.pcbi.1011522)
Supplement: S1 Text — (PDF) [file pcbi.1011522.s002.pdf]

Analytical expression and analysis for P_k

Exact expression

A simple calculation using Eqs. (3-5) of the main text yields

$$P_k = \frac{\omega}{\alpha+\omega}\left(\frac{\alpha}{\alpha+\omega}\right)^k \frac{\gamma(k+1,(\alpha+\omega)\mathcal{T}(L))}{k!} + \frac{(\alpha\mathcal{T}(L))^k}{k!}e^{-(\alpha+\omega)\mathcal{T}(L)}, \quad (1)$$

where $\gamma(r,z)$ is the incomplete gamma function defined by

$$\gamma(r,z) = \int_0^z e^{-t}t^{r-1}\,dt\,. \quad (2)$$

For non-negative integers k, it may be expressed in terms of elementary functions as

$$\frac{\gamma(k+1,z)}{k!} = 1 - e^{-z}\sum_{n=0}^{k}\frac{z^n}{n!} \quad (3)$$

$$= 1 - \frac{z^k e^{-z}}{k!}\sum_{m=0}^{k}\frac{k!}{(k-m)!z^m}. \quad (4)$$

For $k = z \gg 1$, the above approximation needs to be replaced by

$$\frac{\gamma(z+1,z)}{z!} \approx \frac{1}{2}\left(1 - \frac{4}{3\sqrt{2\pi z}}\right). \quad (5)$$

Linear expansion in $\tilde{\omega}$

When $\tilde{\omega}$ is small compared to $\tilde{\alpha}$, we may perform a linear expansion of P_k in $\tilde{\omega}/\tilde{\alpha}$. Using (3) for $\tilde{\alpha} \gg 1$ and $k \ll \tilde{\alpha}$, yields

$$P_k = P_k^\infty + \frac{\tilde{\omega}}{\tilde{\alpha}} + \mathcal{O}\left(\frac{\tilde{\omega}}{\tilde{\alpha}}\right)^2, \quad (6)$$

where

$$P_k^\infty = \frac{\tilde{\alpha}^k e^{-\tilde{\alpha}}}{k!} \quad (7)$$

is the expected Poisson distribution in the zero degradation ($\tilde{\omega} = 0$) or infinite lifetime limit (darkest blue curve in Fig 3 of the main text). For small enough k and large enough $\tilde{\alpha}$, within the biologically accessible window,

$$1 \gg \tilde{\omega} \gg \frac{\tilde{\omega}}{\tilde{\alpha}} \gg P_k^\infty \text{ and } \left(\frac{\tilde{\omega}}{\tilde{\alpha}}\right)^2, \quad (8)$$

first order (in $\tilde{\omega}$) degradation effects dominate and $P_k \approx \tilde{\omega}/\tilde{\alpha}$.

Gaussian approximation for P_k^∞

For $k > 1$ and $\tilde{\alpha} \gg 1$, using the Stirling formula and a saddle point approximation, we have

$$P_k^\infty \approx \frac{\tilde{\alpha}^k k^{-k} e^{k-\tilde{\alpha}}}{\sqrt{2\pi k}} \approx \frac{\exp\left[-(k-\tilde{\alpha}+1/2)^2/(2\tilde{\alpha})\right]}{\sqrt{2\pi\tilde{\alpha}}}. \tag{9}$$

In this limit, P_k^∞ reaches a maximum value given by $1/\sqrt{2\pi\tilde{\alpha}}$ at $k \approx \tilde{\alpha} - 1/2$. This Gaussian approximation qualitatively captures the exponential suppression of k-somes in the absence of degradation when $k \ll \tilde{\alpha} - \sqrt{\tilde{\alpha}}$.

Population inversion

When $\tilde{\omega}$ increases and reaches a crossover value $\tilde{\omega}_{\text{co}}$, the value of P_0 becomes higher than the maximum near $k = \tilde{\alpha}$ which means that low order k-somes take over those with an average number of ribosomes close to $\langle k \rangle_\infty = \tilde{\alpha}$. To approximately determine $\tilde{\omega}_{\text{co}}$, we note that, for finite but not too high degradation, the position of the maximum of P_k stays close to $k \approx \tilde{\alpha}$. We can therefore estimate $\tilde{\omega}_{\text{co}}$ by equating $P_{\tilde{\alpha}}$ with $P_0 \approx \tilde{\omega}/\tilde{\alpha} \ll 1$:

$$P_{\tilde{\alpha}} \approx \left(\frac{\tilde{\omega}}{2\tilde{\alpha}} + \frac{1}{\sqrt{2\pi\tilde{\alpha}}}\right) e^{-\tilde{\omega}} = P_0 \approx \frac{\tilde{\omega}}{\tilde{\alpha}}, \tag{10}$$

where we have used (5) to obtain the approximation for $P_{\tilde{\alpha}}$. Solving (10) for $\tilde{\omega}$ leads to an accurate relation between $\tilde{\omega}_{\text{co}}$ and $\tilde{\alpha}$ valid for $\tilde{\alpha} > 2$:

$$\tilde{\alpha} \approx 2\pi\tilde{\omega}_{\text{co}}^2 \left(e^{\tilde{\omega}_{\text{co}}} - \frac{1}{2}\right)^2. \tag{11}$$

This relation reveals that $\tilde{\omega}_{\text{co}}$ increases only slowly with $\tilde{\alpha}$ (roughly logarithmically) and that $\tilde{\omega}_{\text{co}} \sim 1$ for $10 < \tilde{\alpha} < 100$, which allows one to obtain a simpler but still accurate direct estimate:

$$\tilde{\omega}_{\text{co}} = \ln\left(\sqrt{\frac{\tilde{\alpha}}{2\pi\tilde{\omega}_{\text{co}}}} + \frac{1}{2}\right) \approx \ln\left(\sqrt{\frac{\tilde{\alpha}}{2\pi}} + \frac{1}{2}\right). \tag{12}$$
