# Supplementary material for: Physical modeling of ribosomes along messenger RNA: Estimating kinetic parameters from ribosome profiling experiments using a ballistic model
Source: PLoS Comput Biol. 2023 Oct 20;19(10):e1011522. doi: 10.1371/journal.pcbi.1011522 (PMC10659217; doi:10.1371/journal.pcbi.1011522)
Supplement: S2 Text — (PDF) [file pcbi.1011522.s003.pdf]

Function $f(\tilde{\omega})$: a birth and death approach

We give a heuristic explanation of the roles played by the three parameters α, $\mathcal{T}(L)$ and $\langle\theta\rangle$ in determining the average polysome density. Within the framework of the ballistic model both the inverse of the time necessary to cross the mRNA, $1/\mathcal{T}(L)$, and of the mean lifetime, $1/\langle\theta\rangle$, of the mRNA play the role of effective mortality rates that compensate the initiation (or birth) rate, α, leading to an average number of ribosomes $\langle k\rangle$ on the mRNA. The two mortality rates, $1/\mathcal{T}(L)$ and $1/\langle\theta\rangle = \omega$, are clearly in competition and it is the highest one that primarily determines $\langle k\rangle$. A simple interpolation, which leads to the correct limiting forms for small and large $\tilde{\omega}$ takes the form

$$\frac{1}{\langle k\rangle} \approx \frac{\mathcal{T}(L)^{-1}}{\alpha} + \frac{\langle\theta\rangle^{-1}}{\alpha} = \frac{1}{\tilde{\alpha}}\left(1 + \tilde{\omega}\right).$$
