# Supplementary material for: Physical modeling of ribosomes along messenger RNA: Estimating kinetic parameters from ribosome profiling experiments using a ballistic model
Source: PLoS Comput Biol. 2023 Oct 20;19(10):e1011522. doi: 10.1371/journal.pcbi.1011522 (PMC10659217; doi:10.1371/journal.pcbi.1011522)
Supplement: S3 Text — (PDF) [file pcbi.1011522.s004.pdf]

Gamma function: exact formulas and approximations

Definition and exact formulas

The incomplete Gamma function is defined by [?]

$$\gamma(r,z) = \int_0^z e^{-t} t^{r-1}\, dt \tag{1}$$

For non-negative integers k, it may be expressed in terms of elementary functions as

$$\frac{\gamma(k+1,z)}{k!} = 1 - e^{-z} \sum_{n=0}^{k} \frac{z^n}{n!} \tag{2}$$

$$= 1 - \frac{z^k e^{-z}}{k!} \sum_{m=0}^{k} \frac{k!}{(k-m)! z^m}. \tag{3}$$

Useful approximations

For $k \ll z$, it is a good approximation to keep only the first two terms in the sum of (2):

$$\frac{\gamma(k+1,z)}{k!} \approx 1 - \frac{z^k e^{-z}}{k!} \left(1 + \frac{k}{z}\right), \tag{4}$$

a result that is exact for $k = 0$ and 1. For $k = z \gg 1$, the above approximation needs to be replaced by

$$\frac{\gamma(z+1,z)}{z!} \approx \frac{1}{2} \left(1 - \frac{4}{3\sqrt{2\pi z}}\right). \tag{5}$$
