# Supplementary material for: Physical modeling of ribosomes along messenger RNA: Estimating kinetic parameters from ribosome profiling experiments using a ballistic model
Source: PLoS Comput Biol. 2023 Oct 20;19(10):e1011522. doi: 10.1371/journal.pcbi.1011522 (PMC10659217; doi:10.1371/journal.pcbi.1011522)
Supplement: S4 Text — (PDF) [file pcbi.1011522.s005.pdf]

Transient to stationary ratio for k-some densities

To characterize the whole population of mRNAs measured in Ribo-seq experiments, we split them into two distinct groups according to their age a: mRNAs in the transient regime of filling ($a \leq \mathcal{T}(L)$) and mRNAs in the steady state of translation ($a \geq \mathcal{T}(L)$). This distinction has already been made in the analytical description of the model. We therefore write the k-some density as the sum of two contributions:

$$\rho_k(x) = \mathcal{F}_k(x) + \mathcal{S}_k(x), \tag{1}$$

where $\mathcal{F}_k(x)$ is the density of mRNAs in the transient regime of filling,

$$\mathcal{F}_k(x) = \frac{\tilde{\omega}}{P_k \tilde{p}(x)} \left(\frac{\tilde{\alpha}}{\tilde{\alpha}+\tilde{\omega}}\right)^k \frac{\gamma(k,(\tilde{\alpha}+\tilde{\omega})) - \gamma(k,(\tilde{\alpha}+\tilde{\omega})\tau(x))}{(k-1)!}\,, \tag{2}$$

with $\tau(x) = \mathcal{T}(x)/\mathcal{T}(L)$. $\mathcal{S}_k(x)$ is the density of mRNAs in their steady state of translation,

$$\mathcal{S}_k(x) = \frac{\tilde{\alpha}^k}{P_k\,\tilde{p}(x)} \frac{e^{-(\tilde{\alpha}+\tilde{\omega})}}{(k-1)!}\,. \tag{3}$$

$\mathcal{F}_k(x)$ obviously bears most of the finite mRNA lifetime effects. We therefore choose to quantify the influence of mRNA degradation on k-somes by defining a transient to stationary mRNA density ratio as follows,

$$\mathcal{R}_k(x) = \frac{\mathcal{F}_k(x)}{\mathcal{S}_k(x)} = \frac{\tilde{\omega}}{(\tilde{\alpha}+\tilde{\omega})^k} \left[\gamma(k,\tilde{\alpha}+\tilde{\omega}) - \gamma(k,(\tilde{\alpha}+\tilde{\omega})\tau(x))\right] e^{\tilde{\alpha}+\tilde{\omega}}\,. \tag{4}$$

To simplify the analysis we use its maximal value (achieved at $x = 0$):

$$\mathcal{R}_k(0) = \tilde{\omega} \frac{\gamma(k,\tilde{\omega}+\tilde{\alpha})}{(\tilde{\omega}+\tilde{\alpha})^k} e^{\tilde{\omega}+\tilde{\alpha}}\,. \tag{5}$$

We can measure the effect of finite mRNA lifetime on $\rho_k(x)$ by considering it to manifest itself when $\mathcal{R}_k(0)$ is on the order of unity. The value of $\mathcal{R}_k(0)$ being fixed, exact parametric representations for degradation threshold curves in the $(\tilde{\alpha}, \tilde{\omega})$ parameter space read as follows:

$$\tilde{\omega}_k^{\mathrm{th}}(u) = \mathcal{R}_k(0) \frac{u^k e^{-u}}{\gamma(k,u)} \quad ; \quad \tilde{\alpha}(u) = u - \tilde{\omega}_k^{\mathrm{th}}(u) \tag{6}$$

with $u \geq u_k$ where u_k is the positive solution to $\tilde{\alpha}(u_k) = 0$. These parametric representations are those used in Fig. 1 for $k = 1$ to 4 to display the exact degradation crossover curves (solid lines) for low order k-somes.

When $\tilde{\omega} + \tilde{\alpha} \gg k$ and $\tilde{\alpha} \gg \tilde{\omega}$, reasonable assumptions for many biological systems, for low enough values of k, the threshold degradation rate, $\tilde{\omega}_k^{\mathrm{th}}$, above which a k-some is impacted by degradation simplifies to

$$\tilde{\omega}_k^{\mathrm{th}} \approx \mathcal{R}_k(0) \frac{\tilde{\alpha}^k}{k!} e^{-\tilde{\alpha}}, \quad \text{for}\, k < \tilde{\alpha}\,. \tag{7}$$

This threshold degradation rate depends on the value chosen for $\mathcal{R}_k(0)$. Threshold curves for k-some densities characterized by $\mathcal{R}_k(0) = 1$ are plotted in Fig. 1 with solid lines. Asymptotic expressions provided by (7), starting precisely at the values $\tilde{\alpha} = k$, and displayed as same color dashed lines, confirm their domain of validity. These expressions, which show that $\tilde{\omega}_k^{\text{th}}(\tilde{\alpha})$ is a sharply decreasing function of $\tilde{\alpha}$ for $\tilde{\alpha} > k$, are valid up to relatively high degradation values since for $\tilde{\alpha} = k$, $\tilde{\omega}_k^{\text{th}}(\tilde{\alpha}) \simeq \mathcal{R}_k(0)\sqrt{k/2\pi}$.

We observe in Fig. 1 that, the lower the value of k, the more sensitive k-some densities become to mRNA degradation. The monosome density (blue curve) is by far the most sensitive to degradation and is therefore used in the minimization method exposed in Section 5.3 of the main text to fit the model parameters. Its exact ratio reads

$$\mathcal{R}_1(0) = \frac{\tilde{\omega}}{\tilde{\omega} + \tilde{\alpha}} \left(e^{\tilde{\omega} + \tilde{\alpha}} - 1 \right) \approx \frac{\tilde{\omega}}{\tilde{\alpha} e^{-\tilde{\alpha}}} \,, \tag{8}$$

where the approximation holds for $\tilde{\alpha} \gg 1$ and $\tilde{\omega} \ll 1$.

In Fig. 2, we illustrate the decomposition of $\rho_1(x)$ for the homogeneous (constant $p(x)$) ballistic model. The two regimes displayed in this figure correspond to the values of the parameters used in the upper and intermediate panels of Fig. F4. For this homogeneous ballistic model, the nonlinear contribution to the monosome density only comes from $\mathcal{F}_1(x)$. When the finite lifetime effect is relatively low, $\mathcal{R}_1(0) \sim 1$ (left panel), the density of monosomes in the transient regime of filling, $\mathcal{F}_1(x)$, barely matches in the vicinity of the mRNA entrance that in the steady state of filling, $\mathcal{S}_1$, and stays much lower on the rest of the filament. On the contrary, when the finite lifetime effect is high, $\mathcal{R}_1(0) \sim 10^3$ (right panel), $\mathcal{F}_1(x)$ is large compared to $\mathcal{S}_1$ over the entire filament except at its very end, and the density $\rho_1(x)$ is well approximated by $\mathcal{F}_1(x)$ almost everywhere.

For an illustration of the relevance of this ratio to determine the state of the system, we present a comparison of profiles with different parameters but same $\mathcal{R}_1(0)$ in Fig.3.

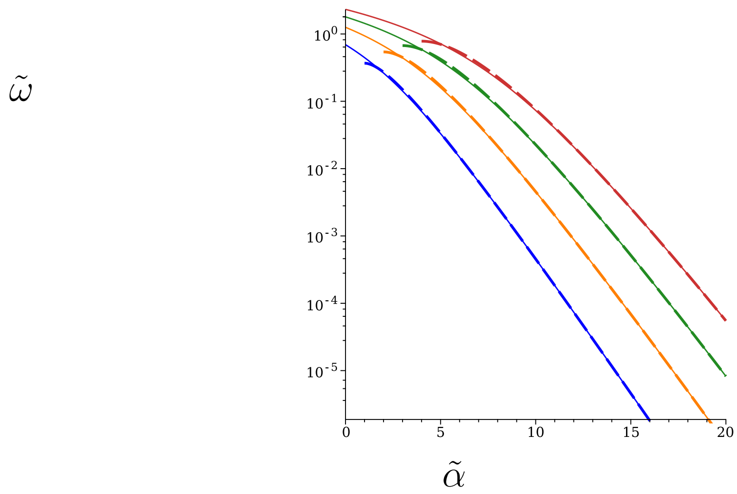

Figure 1: Degradation crossover curves $\tilde{\omega}_k^{\text{th}}(\tilde{\alpha})$ with $\mathcal{R}_k(0) = 1$ for k-some densities obtained from (5) (solid lines: $k = 1$ (blue), $k = 2$ (orange), $k = 3$ (green), $k = 4$ (red)). Dashed lines with same color are the asymptotic expressions given by (7) starting at $\tilde{\alpha} = k$. These curves split the $(\tilde{\alpha}, \tilde{\omega})$ parameter space into two regions: for a given value of k, below the corresponding curve, mRNA degradation barely impacts k-some densities (near the mRNA initiation site) while above, it does more strongly.

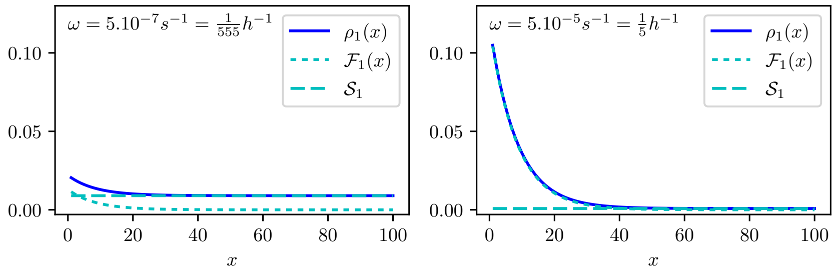

Figure 2: Transient and stationary contributions of the monosome density ($\rho_1(x)$) for the regimes A and B illustrated in Fig. 4 ($\alpha = 0.06s^{-1}$ and $p = 1/2s^{-1}$ for a filament of size $L = 100$ sites (in codon unit). Regime A is on the left, the degradation take the value $\omega = 5.10^{-7}s^{-1}$ ($\tilde{\omega} = 10^{-4}$, $\mathcal{R}_1(0) \approx 1.4$), and for the regime B on the right $\omega = 5.10^{-5}s^{-1}$ ($\tilde{\omega} = 10^{-2}$, $\mathcal{R}_1(0) \approx 1.4.10^2$).

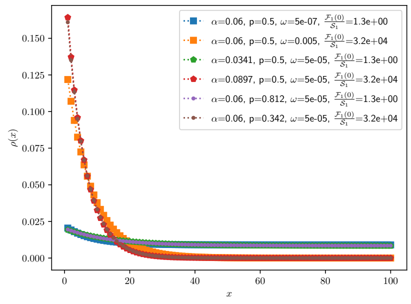

Figure 3: Monosome densities for $\mathcal{F}_1(0)/\mathcal{S}_1 = 1.3$ corresponding to the the low finite lifetime effect on k-somes and for $\mathcal{F}_1(0)/\mathcal{S}_1 = 1.3 \times 10^4$ corresponding to the high finite lifetime effect on k-somes. The regimes can be obtain by different values of α, p and ω as $\mathcal{F}_1(0)/\mathcal{S}_1$ remain constant.

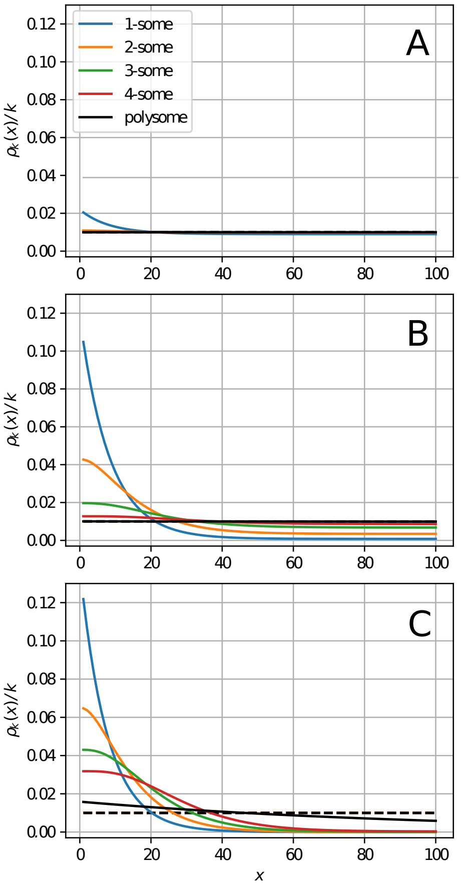

Figure 4: Plot of the normalized (to 1) k-some densities $\bar{\rho}_k$ for $k = 1$ to 4 versus the genomic coordinate x. The corresponding non-normalized data are shown in Fig.4 of the main text). The curves are obtained from Eq.(15) of the main text: monosome (blue), disome (orange), trisome (green) and tetrasome (red). Black curves correspond to polysome densities. Parameters are $\alpha = 0.06s^{-1}$, $p = 1/2s^{-1}$ and $L = 100$ codons ($\tilde{\alpha} = 12$). Solid lines are obtained from E 15 for increasing finite values of ω. (A) $\omega = 5.10^{-7}s^{-1}$ ($\tilde{\omega} = 10^{-4}$), (B) $\omega = 5.10^{-5}s^{-1}$ ($\tilde{\omega} = 10^{-2}$), (C) $\omega = 5.10^{-3}s^{-1}$ ($\tilde{\omega} = 1$). Dashed lines are obtained for $\omega = 0s^{-1}$ (infinite mRNA lifetime). In panels (B-C), the profiles show clearly that near the entrance of the mRNA (over a genomic distance of 30-40 codons) normalized k-some density profiles decrease with increasing k while they increase with k beyond that distance. This crossing of the k-some density curves is another key prediction of the ballistic model in the presence of mRNA degradation. The same crossing is displayed by experimental data as shown in Fig 5 of the main text.
