# Supplementary material for: Physical modeling of ribosomes along messenger RNA: Estimating kinetic parameters from ribosome profiling experiments using a ballistic model
Source: PLoS Comput Biol. 2023 Oct 20;19(10):e1011522. doi: 10.1371/journal.pcbi.1011522 (PMC10659217; doi:10.1371/journal.pcbi.1011522)
Supplement: S5 Text — (PDF) [file pcbi.1011522.s006.pdf]

Discrete approach of the ballistic model

Discrete density: definition and normalisation

The ballistic model presented in the main article has been formulated in continuous space $x \in [0, L]$, where L is the mRNA length in codon units. In the biological realm of mRNA, however, codons are separated units that should be considered as sites forming a lattice with L sites. Ribosome densities (for polysomes and k-somes alike) are then naturally defined on that lattice as discrete quantities. In order to somehow recover that discreteness, we shall simply define the density at site i as the integral of the continuous one over the previous interval $x \in [i-1, i]$, i.e.

$$\rho_i = \int_{i-1}^{i} \rho(x)\, dx\,. \tag{1}$$

The normalisation of that discrete density is therefore the same as its continuous counterpart

$$\sum_{i=1}^{L} \rho_i = \int_0^L \rho(x)\, dx\,. \tag{2}$$

Polysome discrete density

The polysome density normalised to unity is provided in Eq.(18) of the main text. Using (1), we obtain its discrete counterpart,

$$\bar{\rho}_i = \frac{e^{-\omega \mathcal{T}_{i-1}} - e^{-\omega \mathcal{T}_i}}{1 - e^{-\omega \mathcal{T}_L}}\,, \tag{3}$$

where the integrated time taken by a ribosome to go from site 1 to i ($i \in [\![1, L]\!]$) is now:

$$\mathcal{T}_i = \int_0^i \frac{dx}{p(x)} = \sum_{j=1}^{i} \frac{1}{p_j}\,, \tag{4}$$

where $p(x)$ is considered as a piecewise constant function: $p(x) = p_i$ for $x \in]i-1, i]$.

k-some discrete densities

The expression of the k-some density at site i is given by

$$\rho_{k;i} = \left(\frac{\tilde{\alpha}}{\tilde{\alpha}+\tilde{\omega}}\right)^k \frac{\tilde{\omega}}{P_k (k-1)!}\left(\frac{\gamma(k,(\tilde{\alpha}+\tilde{\omega}))}{\tilde{p}_i} - (\mathcal{I}_i - \mathcal{I}_{i-1})\right) + \frac{1}{\tilde{p}_i P_k}\frac{\tilde{\alpha}^k}{(k-1)!} e^{-(\tilde{\alpha}+\tilde{\omega})}\,, \tag{5}$$

where

$$\mathcal{I}_i = \tau_i \gamma(k, (\tilde{\alpha}+\tilde{\omega})\tau_i) - \frac{\gamma(k+1, (\tilde{\alpha}+\tilde{\omega})\tau_i)}{\tilde{\alpha}+\tilde{\omega}}\,. \tag{6}$$

with $\tilde{\alpha} = \alpha \mathcal{T}_L$, $\tilde{\omega} = \omega \mathcal{T}_L$, $\tilde{p} = p \mathcal{T}_L$ and $\tau_i = \mathcal{T}_i / \mathcal{T}_L$. The expression of P_k remains the same as in the continuous case. In the infinite mRNA lifetime limit ($\omega \to 0$)

expressions simplify: the k-some distribution becomes $P_k^\infty = \tilde{\alpha}^k e^{-\tilde{\alpha}}/k!$, the k-some density, $\rho_{k;i}^\infty = k/\tilde{p}_i$ while the polysome density reads, $\rho_i^\infty = \alpha/p_i$.
