# Supplementary material for: Physical modeling of ribosomes along messenger RNA: Estimating kinetic parameters from ribosome profiling experiments using a ballistic model
Source: PLoS Comput Biol. 2023 Oct 20;19(10):e1011522. doi: 10.1371/journal.pcbi.1011522 (PMC10659217; doi:10.1371/journal.pcbi.1011522)
Supplement: S6 Text — (PDF) [file pcbi.1011522.s007.pdf]

# Detailed analysis of the $k$-some densities

## Linear expansion in $\tilde{\omega}$ and crossover positions $x_k^{\rm co}$

As it may be seen in Fig. 4 in the main text, for nonzero $\tilde{\omega}$, $k$-some density profiles cross their flat infinite lifetime ($\tilde{\omega} = 0$) counterpart at a genomic coordinate that we shall name $x_k^{\rm co}$: the density is higher for $x < x_k^{\rm co}$ and becomes lower for $x > x_k^{\rm co}$. We moreover observe in the same figure that, for $1 \le k \le 4$, these crossover positions $x_k^{\rm co}$ are to a good approximation independent of $\tilde{\omega}$. To explain this feature, we expand the $k$-some density Eq.(15) in the main text linearly in $\tilde{\omega}$ and find

$$\rho_k(x) = \frac{k}{\tilde{p}(x)} \left[ 1 + \frac{\tilde{\omega}}{k P_k^\infty} \left( \frac{\gamma(k,\tilde{\alpha})}{(k-1)!} - \frac{\gamma(k+1,\tilde{\alpha})}{\tilde{\alpha}(k-1)!} - \frac{\gamma\big(k,\tilde{\alpha}\tau(x)\big)}{(k-1)!} + \mathcal{O}\left(\frac{\tilde{\omega}}{\tilde{\alpha}}\right) \right) \right], \tag{1}$$

Within this exact linear expansion, the first order correction has to vanish at $x_k^{\rm co}$. As $\tilde{\omega}$ is typically much smaller than $\tilde{\alpha}$ for biologically relevant values, we therefore expect $x_k^{\rm co}$ to be essentially independent of $\tilde{\omega}$, indeed.

## Position dependent sensitivity to degradation

We come here to the subtle point that $k$-some density profiles are not uniformly sensitive to degradation. Close to the initiation site ($x = 0$), the linear expansion (1) allows us to understand how the $k$-some density is impacted by degradation: for $x \to 0$, the corrective term to the infinite lifetime limit, $\rho_k^\infty = k/\tilde{p}(x)$, is governed by the natural effective parameter $\tilde{\omega}/(kP_k^\infty)$: the term in large parentheses in (1) reduces to 1 for $\tilde{\alpha} \gg k$, a limit that we have used to obtain the relative density difference given in Eq. (17) in the main text.

When $x \to L$ on the other hand, it is better to rewrite $\rho_k(x)$ as

$$\rho_k(x) = \frac{k}{\tilde{p}(x)} \left[ 1 - \frac{\tilde{\omega}}{\tilde{\alpha} P_k^\infty} \left( \frac{\gamma(k+1,\tilde{\alpha})}{k!} + \tilde{\alpha} \left\{ \frac{\gamma\big(k,\tilde{\alpha}\tau(x)\big)}{k!} - \frac{\gamma(k,\tilde{\alpha})}{k!} \right\} + \mathcal{O}\left(\frac{\tilde{\omega}}{\tilde{\alpha}}\right) \right) \right], \tag{2}$$

for, in that limit, $\tau(x) \to 1$ and, for $\tilde{\alpha} \gg k$, the term in large parentheses again tends to 1. This clearly shows that the natural corrective parameter is now $\tilde{\omega}/(\tilde{\alpha}P_k^\infty)$ which is different from the former one by a factor of $k/\tilde{\alpha}$.

We have then basically shown that for $x < x_k^{\rm co}$, $\tilde{\omega}/(kP_k^\infty)$ is the natural effective parameter probing both the effects of the finite mRNA lifetime and of the initiation rate on the $k$-some density while for $x > x_k^{\rm co}$, it is rather $\tilde{\omega}/(\tilde{\alpha}P_k^\infty)$. For $k \sim 1$ and $\tilde{\alpha} \sim 10$, an order of magnitude separates these effective parameters.

## Monosome density

Let us consider the density of monosomes ($k = 1$) in more detail, as it is the most sensitive to $\tilde{\omega}$. Upon using Eq. (15) and

$$\frac{\gamma(k+1,z)}{k!} = 1 - e^{-z}\sum_{n=0}^{k}\frac{z^n}{n!} \tag{3}$$

we obtain

$$\rho_1(x) = \frac{(\tilde{\omega}+\tilde{\alpha})\left(\tilde{\alpha}+\tilde{\omega}e^{(\tilde{\omega}+\tilde{\alpha})[1-\tau(x)]}\right)}{\tilde{p}(x)\left[\tilde{\alpha}(\tilde{\omega}+\tilde{\alpha})+\tilde{\omega}(e^{\tilde{\omega}+\tilde{\alpha}}-1)\right]}\,. \tag{4}$$

Let us first observe that this density is indeed globally exponentially decaying as $x$ (i.e. $\tau(x)$) increases. The crossover position $x_1^{\text{co}}$ is determined from the equation $\rho_1(x_1^{\text{co}}) = \rho_1^\infty = 1/\tilde{p}(x)$. This yields the exact solution:

$$\tau(x_1^{\text{co}}) = \frac{\ln\left(\frac{\tilde{\alpha}+\tilde{\omega}}{1-e^{-(\tilde{\alpha}+\tilde{\omega})}}\right)}{\tilde{\alpha}+\tilde{\omega}} \approx \frac{\ln\left(\frac{\tilde{\alpha}}{1-e^{-\tilde{\alpha}}}\right)}{\tilde{\alpha}} \tag{5}$$

where the approximation is valid for $\tilde{\alpha} \gg \tilde{\omega}$. Note that $x_1^{\text{co}}$ is a function of the parameter $\tilde{\alpha}+\tilde{\omega}$ only. This is not exactly true anymore for $k > 1$. For a codon independent elongation rate, $\tilde{p}(x) = \tilde{p}$, $\tau(x) = x/L$ and expression (5) provides an explicit expression for the crossover position. Now, from (2), the linear expansion of the monosome density in $\omega$ reads

$$\rho_1(x) = \frac{1}{\tilde{p}(x)}\left[1 - \frac{\tilde{\omega}}{\tilde{\alpha}P_1^\infty}\left(1 - \tilde{\alpha}e^{-\tilde{\alpha}\tau(x)} - e^{-\tilde{\alpha}} + \mathcal{O}\left(\frac{\tilde{\omega}}{\tilde{\alpha}}\right)\right)\right]\,. \tag{6}$$

To this order in $\tilde{\omega}$, we find the same expression for $x_1^{\text{co}}$ as the approximation provided in (5). This result shows that $x_1^{\text{co}}$ decreases with increasing $\tilde{\alpha}$. For $\tilde{\alpha} = 12$ and a constant elongation rate as in Fig.4 in the main text, we obtain $x_1^{\text{co}} \simeq L\ln(\tilde{\alpha})/\tilde{\alpha} \simeq 20.7$, in perfect agreement with the value observed on the figure. For the rather high value $\tilde{\omega} = 1$, replacing $\tilde{\alpha}$ by $\tilde{\alpha}+\tilde{\omega}$ in the last approximation leads to $x_1^{\text{co}} \simeq 19.7$, a slightly lower value of the crossover position that can also be observed on the panel C of Fig. 4 in the main text.

Finally, in this simple but important monosome case, we can illustrate the difference in sensitivity to degradation due to position by evaluating $\rho_1(x)$ at $x = 0$ and $L$:

$$\rho_1(0) = \frac{1}{\tilde{p}(0)}\left[1 + \frac{\tilde{\omega}}{P_1^\infty}\left(1 - \frac{1}{\tilde{\alpha}}\left(1 - e^{-\tilde{\alpha}}\right) + \mathcal{O}\left(\frac{\tilde{\omega}}{\tilde{\alpha}}\right)\right)\right] \tag{7}$$

and

$$\rho_1(L) = \frac{1}{\tilde{p}(L)}\left[1 - \frac{\tilde{\omega}}{\tilde{\alpha}P_1^\infty}\left(1 - e^{-\tilde{\alpha}}\left(\tilde{\alpha}+1\right) + \mathcal{O}\left(\frac{\tilde{\omega}}{\tilde{\alpha}}\right)\right)\right]\,, \tag{8}$$

where $P_1^\infty = \tilde{\alpha}e^{-\tilde{\alpha}}$. Clearly, $\rho_1(0) > 1/\tilde{p}(0)$ and $\rho_1(L) < 1/\tilde{p}(L)$ with $\rho_1(0)$ increasing and $\rho_1(L)$ decreasing with increasing $\tilde{\alpha}$.

From this expression, we see that for monosomes the crossover between the regimes of low and high degradation near the beginning of the mRNA depends on the parameter $\tilde{\omega}/(\tilde{\alpha}e^{-\tilde{\alpha}})$ in contradistinction to the polysomes for which only $\tilde{\omega}$ intervenes and to $P_1$ and $\rho_1(L)$ for which $\tilde{\omega}/(\tilde{\alpha}^2e^{-\tilde{\alpha}})$ intervenes instead.

For typical biological value $\tilde{\alpha} \sim 5$ to 10, the threshold value of the degradation rate for monosome densities near the mRNA entrance, $\tilde{\omega} \sim P_1^\infty = \tilde{\alpha} e^{-\tilde{\alpha}}$, then decreases by a factor 30 to 2000 compared to that of polysomes ($\tilde{\omega} \sim 1$). Sensitivities to degradation then clearly differ by 1 to 3 orders of magnitude.
